# *N*′-(5-Bromo-2-hy­droxy­benzyl­idene)-3-nitro­benzohydrazide methanol mono­solvate

**DOI:** 10.1107/S1600536811042553

**Published:** 2011-10-22

**Authors:** Chun-Bao Tang

**Affiliations:** a Department of Chemistry, Jiaying University, Meizhou 514015, People’s Republic of China

## Abstract

In the title compound, C_14_H_10_BrN_3_O_4_·CH_4_O, the dihedral angle between the two benzene rings in the hydrazone mol­ecule is 5.8 (3)° and an intra­molecular O—H⋯N hydrogen bond generates an *S*(6) ring motif. An O—H⋯O hydrogen bond occurs between the hydrazone mol­ecule and the methanol solvent mol­ecule. In the crystal, the components are linked by inter­molecular N—H⋯O hydrogen bonds, forming chains along the *a* axis.

## Related literature

For general background to hydrazones, see: Rasras *et al.* (2010 ▶); Pyta *et al.* (2010 ▶); Angelusiu *et al.* (2010 ▶). For related structures, see: Fun *et al.* (2008 ▶); Singh & Singh (2010 ▶); Ahmad *et al.* (2010 ▶); Tang (2010 ▶, 2011 ▶). For reference bond-length data, see: Allen *et al.* (1987 ▶) and for hydrogen-bond motifs, see: Bernstein *et al.* (1995 ▶).
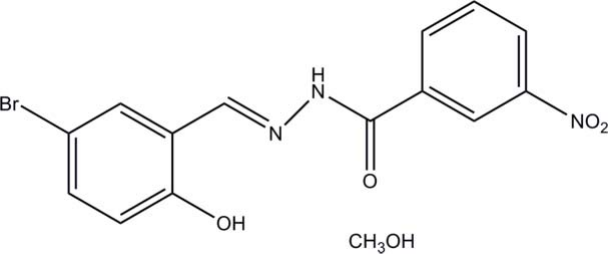


## Experimental

### 

#### Crystal data

C_14_H_10_BrN_3_O_4_·CH_4_O
*M* *_r_* = 396.20
Triclinic, 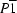

*a* = 6.701 (2) Å
*b* = 9.492 (3) Å
*c* = 13.011 (3) Å
α = 105.866 (2)°
β = 92.535 (2)°
γ = 94.496 (2)°
*V* = 791.7 (4) Å^3^
*Z* = 2
Mo *K*α radiation
μ = 2.63 mm^−1^
*T* = 298 K
0.13 × 0.12 × 0.10 mm

#### Data collection

Bruker SMART CCD area-detector diffractometer
Absorption correction: multi-scan (*SADABS*; Sheldrick, 1996 ▶) *T*_min_ = 0.726, *T*_max_ = 0.779
6325 measured reflections
3356 independent reflections
1142 reflections with *I* > 2σ(*I*)
*R*_int_ = 0.109

#### Refinement

*R*[*F* ^2^ > 2σ(*F* ^2^)] = 0.073
*wR*(*F* ^2^) = 0.236
*S* = 0.93
3356 reflections
223 parameters
1 restraint
H atoms treated by a mixture of independent and constrained refinement
Δρ_max_ = 0.56 e Å^−3^
Δρ_min_ = −0.83 e Å^−3^

### 

Data collection: *SMART* (Bruker, 2002 ▶); cell refinement: *SAINT* (Bruker, 2002 ▶); data reduction: *SAINT*; program(s) used to solve structure: *SHELXS97* (Sheldrick, 2008 ▶); program(s) used to refine structure: *SHELXL97* (Sheldrick, 2008 ▶); molecular graphics: *SHELXTL* (Sheldrick, 2008 ▶); software used to prepare material for publication: *SHELXTL*.

## Supplementary Material

Crystal structure: contains datablock(s) global, I. DOI: 10.1107/S1600536811042553/qm2037sup1.cif

Structure factors: contains datablock(s) I. DOI: 10.1107/S1600536811042553/qm2037Isup2.hkl

Supplementary material file. DOI: 10.1107/S1600536811042553/qm2037Isup3.cml

Additional supplementary materials:  crystallographic information; 3D view; checkCIF report

## Figures and Tables

**Table 1:** Hydrogen-bond geometry (Å, °)

*D*—H⋯*A*	*D*—H	H⋯*A*	*D*⋯*A*	*D*—H⋯*A*
N2—H2⋯O5^i^	0.90 (1)	2.04 (5)	2.854 (10)	150 (9)
O5—H5⋯O2	0.82	1.90	2.701 (10)	166
O1—H1⋯N1	0.82	1.99	2.700 (10)	144

Symmetry code: (i) 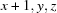.

**Fig. 1.**
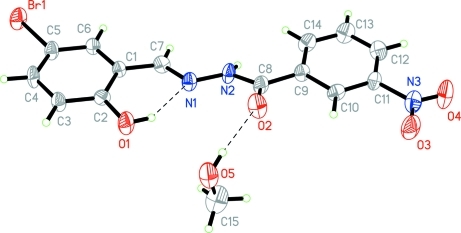
The molecular structure of the compound, showing the atom-numbering scheme. Displacement ellipsoids are drawn at the 30% probability level. Hydrogen atoms are shown as spheres of arbitrary radius and hydrogen bonds are drawn as dashed lines.

**Fig. 2.**
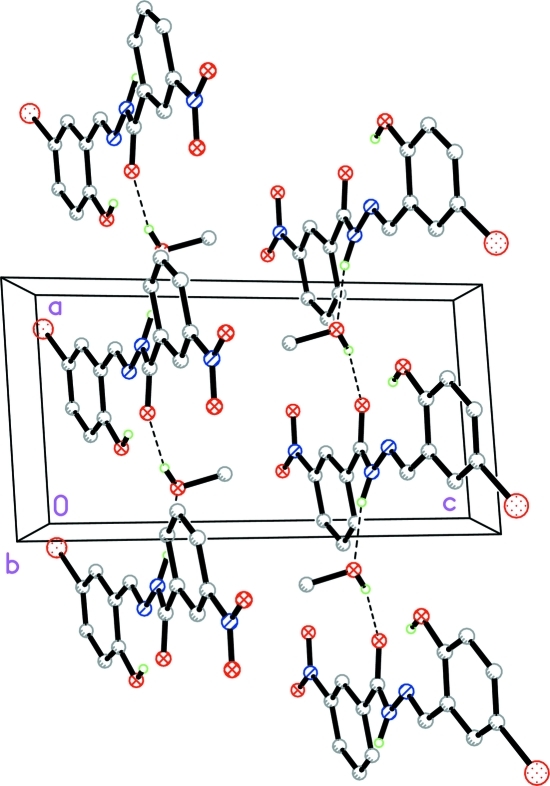
Molecular packing of the title compound, with hydrogen bonds shown as dashed lines.

## supplementary crystallographic information

### Comment

Hydrazone compounds have received much attention in biological and structural chemistry in the last few years (Rasras *et al.*, 2010; Pyta *et al.*, 2010; Angelusiu *et al.*, 2010; Fun *et al.*, 2008; Singh & Singh, 2010; Ahmad *et al.*, 2010). In the present paper, the author reports the crystal structure of the title new hydrazone compound (Fig. 1).

The compound contains a hydrazone molecule and a methanol molecule of crystallization. The dihedral angle between the two benzene rings in the hydrazone molecule is 5.8 (3)°. An intramolecular O—H···N hydrogen bond generates a S(6) ring motif in the hydrazone molecule (Bernstein *et al.*, 1995). Bond lengths in the compound are normal (Allen *et al.*, 1987) and comparable to those in the similar compounds the author has reported previously (Tang, 2010; Tang, 2011). In the crystal structure, the hydrazone molecules are linked by the methanol molecules through intermolecular N—H···O hydrogen bonds (Table 1), forming chains along the *a* axis (Fig. 2).

### Experimental

5-Bromo-2-hydroxybenzaldehyde (0.1 mmol, 20.1 mg) and 3-nitrobenzohydrazide (0.1 mmol, 18.1 mg) were dissolved in methanol (20 ml). The mixture was stirred at reflux for 10 min to give a clear yellow solution. Yellow needle-shaped crystals of the compound were formed by slow evaporation of the solvent over several days.

### Refinement

The amino H atom was located in a difference Fourier map and refined isotropically, with the N—H distance restrained to 0.90 (1) Å [*U*_iso_(H) = 0.08 Å^2^]. Other H atoms were constrained to ideal geometries and refined as riding, with Csp^2^—H = 0.93 Å, C(methyl)—H = 0.96 Å, and O—H = 0.82 Å; *U*_iso_(H) = 1.2*U*_eq_(C) and 1.5*U*_eq_(O and C_methyl_).

### Crystal data

**Table:**

C_14_H_10_BrN_3_O_4_·CH_4_O	*Z* = 2
*M**_r_* = 396.20	*F*(000) = 400
Triclinic, *P*1	*D*_x_ = 1.662 Mg m^−^^3^
*a* = 6.701 (2) Å	Mo *K*α radiation, λ = 0.71073 Å
*b* = 9.492 (3) Å	Cell parameters from 850 reflections
*c* = 13.011 (3) Å	θ = 2.6–24.3°
α = 105.866 (2)°	µ = 2.63 mm^−^^1^
β = 92.535 (2)°	*T* = 298 K
γ = 94.496 (2)°	Cut from needle, yellow
*V* = 791.7 (4) Å^3^	0.13 × 0.12 × 0.10 mm

### Data collection

**Table:**

Bruker SMART CCD area-detector diffractometer	3356 independent reflections
Radiation source: fine-focus sealed tube	1142 reflections with *I* > 2σ(*I*)
graphite	*R*_int_ = 0.109
ω scans	θ_max_ = 27.0°, θ_min_ = 2.2°
Absorption correction: multi-scan (*SADABS*; Sheldrick, 1996)	*h* = −8→8
*T*_min_ = 0.726, *T*_max_ = 0.779	*k* = −12→11
6325 measured reflections	*l* = −16→16

### Refinement

**Table:**

Refinement on *F*^2^	Primary atom site location: structure-invariant direct methods
Least-squares matrix: full	Secondary atom site location: difference Fourier map
*R*[*F*^2^ > 2σ(*F*^2^)] = 0.073	Hydrogen site location: inferred from neighbouring sites
*wR*(*F*^2^) = 0.236	H atoms treated by a mixture of independent and constrained refinement
*S* = 0.93	*w* = 1/[σ^2^(*F*_o_^2^) + (0.0975*P*)^2^] where *P* = (*F*_o_^2^ + 2*F*_c_^2^)/3
3356 reflections	(Δ/σ)_max_ < 0.001
223 parameters	Δρ_max_ = 0.56 e Å^−^^3^
1 restraint	Δρ_min_ = −0.83 e Å^−^^3^

### Special details

**Table:**

Geometry. All esds (except the esd in the dihedral angle between two l.s. planes) are estimated using the full covariance matrix. The cell esds are taken into account individually in the estimation of esds in distances, angles and torsion angles; correlations between esds in cell parameters are only used when they are defined by crystal symmetry. An approximate (isotropic) treatment of cell esds is used for estimating esds involving l.s. planes.
Refinement. Refinement of F^2^ against ALL reflections. The weighted R-factor wR and goodness of fit S are based on F^2^, conventional R-factors R are based on F, with F set to zero for negative F^2^. The threshold expression of F^2^ > 2sigma(F^2^) is used only for calculating R-factors(gt) etc. and is not relevant to the choice of reflections for refinement. R-factors based on F^2^ are statistically about twice as large as those based on F, and R- factors based on ALL data will be even larger.

### Fractional atomic coordinates and isotropic or equivalent isotropic displacement parameters (Å^2^)

**Table:**

	*x*	*y*	*z*	*U*_iso_*/*U*_eq_	
Br1	0.86686 (17)	−0.35734 (12)	−0.00940 (10)	0.0719 (6)	
N1	0.6581 (10)	0.3028 (8)	0.2164 (6)	0.048 (2)	
N2	0.7644 (11)	0.4381 (8)	0.2533 (7)	0.053 (2)	
N3	0.6919 (17)	1.0932 (11)	0.4149 (7)	0.065 (3)	
O1	0.3298 (9)	0.1075 (7)	0.1801 (6)	0.065 (2)	
H1	0.3900	0.1899	0.2010	0.097*	
O2	0.4839 (9)	0.5566 (7)	0.2601 (7)	0.075 (2)	
O3	0.5095 (13)	1.0768 (8)	0.4134 (7)	0.083 (3)	
O4	0.7862 (12)	1.2078 (9)	0.4478 (7)	0.095 (3)	
O5	0.1684 (10)	0.3998 (8)	0.3110 (8)	0.080 (2)	
H5	0.2535	0.4451	0.2858	0.119*	
C1	0.6692 (13)	0.0452 (9)	0.1409 (7)	0.040 (2)	
C2	0.4602 (13)	0.0083 (10)	0.1402 (7)	0.045 (3)	
C3	0.3811 (13)	−0.1355 (10)	0.0995 (8)	0.052 (3)	
H3	0.2446	−0.1599	0.1019	0.062*	
C4	0.5030 (15)	−0.2441 (10)	0.0548 (8)	0.058 (3)	
H4	0.4491	−0.3409	0.0255	0.069*	
C5	0.7013 (14)	−0.2068 (10)	0.0547 (8)	0.051 (3)	
C6	0.7845 (13)	−0.0647 (10)	0.0954 (7)	0.045 (3)	
H6	0.9214	−0.0428	0.0920	0.054*	
C7	0.7583 (13)	0.1925 (10)	0.1837 (8)	0.049 (3)	
H7	0.8975	0.2089	0.1880	0.058*	
C8	0.6684 (15)	0.5605 (10)	0.2710 (8)	0.050 (3)	
C9	0.7917 (13)	0.7039 (10)	0.3036 (8)	0.047 (3)	
C10	0.6934 (13)	0.8259 (11)	0.3455 (7)	0.048 (3)	
H10	0.5569	0.8164	0.3553	0.058*	
C11	0.7981 (15)	0.9610 (10)	0.3723 (8)	0.050 (3)	
C12	1.0014 (16)	0.9800 (12)	0.3593 (8)	0.061 (3)	
H12	1.0697	1.0735	0.3782	0.073*	
C13	1.0973 (15)	0.8607 (13)	0.3191 (9)	0.069 (3)	
H13	1.2341	0.8716	0.3102	0.082*	
C14	0.9956 (14)	0.7203 (12)	0.2903 (8)	0.060 (3)	
H14	1.0640	0.6382	0.2623	0.071*	
C15	0.2258 (18)	0.4128 (14)	0.4176 (12)	0.100 (5)	
H15A	0.2783	0.5124	0.4524	0.149*	
H15B	0.3272	0.3478	0.4208	0.149*	
H15C	0.1116	0.3873	0.4530	0.149*	
H2	0.893 (5)	0.462 (11)	0.280 (8)	0.080*	

### Atomic displacement parameters (Å^2^)

**Table:**

	*U*^11^	*U*^22^	*U*^33^	*U*^12^	*U*^13^	*U*^23^
Br1	0.0633 (8)	0.0472 (7)	0.0991 (11)	0.0263 (5)	0.0049 (6)	0.0041 (6)
N1	0.037 (5)	0.039 (5)	0.066 (6)	0.010 (4)	−0.003 (4)	0.010 (4)
N2	0.037 (5)	0.034 (5)	0.079 (6)	0.006 (4)	−0.003 (4)	−0.001 (4)
N3	0.078 (7)	0.050 (6)	0.065 (7)	0.014 (6)	−0.008 (6)	0.011 (5)
O1	0.046 (4)	0.040 (4)	0.105 (6)	0.014 (3)	0.000 (4)	0.011 (4)
O2	0.027 (4)	0.058 (5)	0.138 (7)	0.009 (3)	−0.002 (4)	0.027 (5)
O3	0.072 (6)	0.063 (5)	0.116 (7)	0.032 (4)	0.010 (5)	0.018 (5)
O4	0.089 (6)	0.045 (5)	0.133 (8)	0.013 (5)	−0.015 (5)	−0.003 (5)
O5	0.041 (5)	0.057 (5)	0.135 (8)	0.006 (4)	−0.005 (5)	0.019 (5)
C1	0.033 (5)	0.035 (6)	0.053 (7)	0.006 (4)	0.000 (5)	0.013 (5)
C2	0.038 (6)	0.041 (6)	0.061 (7)	0.019 (5)	0.014 (5)	0.019 (5)
C3	0.034 (5)	0.037 (6)	0.080 (8)	0.003 (5)	0.001 (5)	0.009 (5)
C4	0.060 (7)	0.024 (5)	0.085 (9)	0.004 (5)	−0.008 (6)	0.009 (5)
C5	0.040 (6)	0.039 (6)	0.068 (8)	0.006 (5)	−0.003 (5)	0.005 (5)
C6	0.030 (5)	0.041 (6)	0.061 (7)	0.006 (4)	0.002 (5)	0.009 (5)
C7	0.033 (5)	0.052 (7)	0.064 (7)	0.012 (5)	−0.001 (5)	0.020 (6)
C8	0.045 (7)	0.042 (6)	0.065 (8)	0.016 (5)	0.003 (5)	0.014 (5)
C9	0.034 (6)	0.044 (6)	0.064 (7)	0.015 (5)	0.000 (5)	0.017 (5)
C10	0.034 (5)	0.059 (7)	0.055 (7)	0.018 (5)	0.002 (5)	0.017 (5)
C11	0.056 (7)	0.036 (6)	0.051 (7)	0.004 (5)	−0.004 (5)	0.002 (5)
C12	0.058 (8)	0.057 (7)	0.063 (8)	−0.002 (6)	−0.011 (6)	0.016 (6)
C13	0.039 (6)	0.072 (8)	0.088 (9)	0.011 (6)	−0.005 (6)	0.011 (7)
C14	0.035 (6)	0.064 (8)	0.069 (8)	0.006 (5)	−0.002 (5)	0.001 (6)
C15	0.087 (10)	0.090 (10)	0.109 (12)	0.017 (8)	−0.023 (9)	0.008 (9)

### Geometric parameters (Å, °)

**Table:**

Br1—C5	1.910 (9)	C4—C5	1.348 (12)
N1—C7	1.273 (10)	C4—H4	0.9300
N1—N2	1.370 (10)	C5—C6	1.370 (11)
N2—C8	1.342 (11)	C6—H6	0.9300
N2—H2	0.901 (10)	C7—H7	0.9300
N3—O4	1.175 (10)	C8—C9	1.481 (13)
N3—O3	1.219 (10)	C9—C10	1.370 (12)
N3—C11	1.477 (12)	C9—C14	1.387 (12)
O1—C2	1.349 (9)	C10—C11	1.360 (12)
O1—H1	0.8200	C10—H10	0.9300
O2—C8	1.235 (10)	C11—C12	1.384 (13)
O5—C15	1.392 (13)	C12—C13	1.338 (13)
O5—H5	0.8200	C12—H12	0.9300
C1—C6	1.366 (11)	C13—C14	1.393 (13)
C1—C2	1.416 (12)	C13—H13	0.9300
C1—C7	1.427 (12)	C14—H14	0.9300
C2—C3	1.375 (12)	C15—H15A	0.9600
C3—C4	1.383 (12)	C15—H15B	0.9600
C3—H3	0.9300	C15—H15C	0.9600
			
C7—N1—N2	117.1 (8)	C1—C7—H7	118.2
C8—N2—N1	119.9 (8)	O2—C8—N2	122.4 (9)
C8—N2—H2	109 (7)	O2—C8—C9	119.9 (8)
N1—N2—H2	130 (7)	N2—C8—C9	117.7 (8)
O4—N3—O3	123.5 (10)	C10—C9—C14	119.5 (9)
O4—N3—C11	118.9 (10)	C10—C9—C8	116.8 (8)
O3—N3—C11	117.6 (9)	C14—C9—C8	123.7 (9)
C2—O1—H1	109.5	C11—C10—C9	119.1 (9)
C15—O5—H5	109.5	C11—C10—H10	120.4
C6—C1—C2	117.8 (8)	C9—C10—H10	120.4
C6—C1—C7	120.2 (8)	C10—C11—C12	122.3 (9)
C2—C1—C7	122.0 (8)	C10—C11—N3	119.4 (9)
O1—C2—C3	116.6 (8)	C12—C11—N3	118.3 (9)
O1—C2—C1	123.4 (8)	C13—C12—C11	118.4 (10)
C3—C2—C1	120.0 (8)	C13—C12—H12	120.8
C2—C3—C4	120.5 (9)	C11—C12—H12	120.8
C2—C3—H3	119.7	C12—C13—C14	121.1 (10)
C4—C3—H3	119.7	C12—C13—H13	119.5
C5—C4—C3	118.7 (9)	C14—C13—H13	119.5
C5—C4—H4	120.6	C9—C14—C13	119.5 (10)
C3—C4—H4	120.6	C9—C14—H14	120.2
C4—C5—C6	122.0 (9)	C13—C14—H14	120.2
C4—C5—Br1	118.2 (7)	O5—C15—H15A	109.5
C6—C5—Br1	119.7 (7)	O5—C15—H15B	109.5
C1—C6—C5	120.9 (8)	H15A—C15—H15B	109.5
C1—C6—H6	119.6	O5—C15—H15C	109.5
C5—C6—H6	119.6	H15A—C15—H15C	109.5
N1—C7—C1	123.7 (8)	H15B—C15—H15C	109.5
N1—C7—H7	118.2		

### Hydrogen-bond geometry (Å, °)

**Table:**

*D*—H···*A*	*D*—H	H···*A*	*D*···*A*	*D*—H···*A*
N2—H2···O5^i^	0.90 (1)	2.04 (5)	2.854 (10)	150 (9)
O5—H5···O2	0.82	1.90	2.701 (10)	166.
O1—H1···N1	0.82	1.99	2.700 (10)	144.

Symmetry codes: (i) *x*+1, *y*, *z*.
